# Targeting NF-κB-Inducing Kinase (NIK) in Immunity, Inflammation, and Cancer

**DOI:** 10.3390/ijms21228470

**Published:** 2020-11-11

**Authors:** Kathryn M. Pflug, Raquel Sitcheran

**Affiliations:** 1 Interdisciplinary Program in Genetics, Texas A&M University, College Station, TX 77843, USA; kpflug@tamu.edu; 2 Department of Molecular & Cellular Medicine, Texas A&M University Health Science Center, Bryan, TX 77002, USA

**Keywords:** NF-κB-inducing kinase, NIK, Map3k14, NF-κB, immunity, inflammatory disease, cancer, therapeutics, small-molecule inhibitors

## Abstract

NF-κB-inducing kinase (NIK), the essential upstream kinase, which regulates activation of the noncanonical NF-κB pathway, has important roles in regulating immunity and inflammation. In addition, NIK is vital for maintaining cellular health through its control of fundamental cellular processes, including differentiation, growth, and cell survival. As such aberrant expression or regulation of NIK is associated with several disease states. For example, loss of NIK leads to severe immune defects, while the overexpression of NIK is observed in inflammatory diseases, metabolic disorders, and the development and progression of cancer. This review discusses recent studies investigating the therapeutic potential of NIK inhibitors in various diseases.

## 1. Introduction

### 1.1. NF-κB Signaling

**Figure 1:**
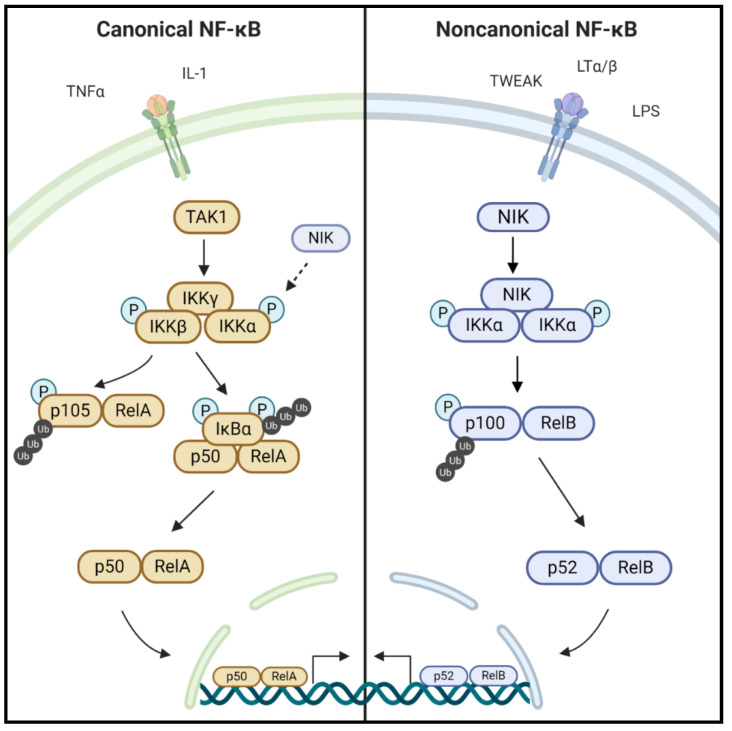
Nuclear factor-kappa light chain enhancer of activated B cells (NF-κB) pathways. NF-κB pathways are stimulated by various cytokines, bacteria, or viruses on different receptors. Activation of the canonical NF-κB pathway leads to various kinases, including transforming growth factor beta-activated kinase 1 (TAK1), phosphorylating the inhibitor of kappa B kinase (IKK) complex preferentially activating IKKβ. Inhibitory kappa B kinase beta (IKKβ) then phosphorylates p105 bound to RelA targeting p105 for partial proteasomal degradation into p50. Additionally, IKKβ phosphorylates IκBα targeting it for proteasomal degradation allowing p50-RelA to translocate to the nucleus. Activation of the noncanonical NF-κB pathway leads to NF-κB-inducing kinase (NIK) activation and subsequent phosphorylation of IKKα. Inhibitory kappa B kinase alpha (IKKα) then phosphorylates p100 marking it for partial proteasomal degradation into p52. RelB bound p52 is then translocated to the nucleus to regulate gene transcription. NF-κB-inducing kinase (NIK) is also able to facilitate canonical NF-κB pathway activation through phosphorylation of the IKKα subunit.

Nuclear factor-kappa light chain enhancer of activated B cells (NF-κB) refers to a family of evolutionarily conserved transcription factors that regulate a wide range of biological processes, including cell growth and survival to immunity and inflammation [1]. In mammals, there are five NF-κB family members: RelA (p65), RelB, c-Rel, NF-κB1 (p105/p50) and NF-κB2 (p100/p52). Activation of NF-κB occurs through two distinct signaling pathways, referred to as the canonical and noncanonical NF-κB pathways, and is often triggered by inflammatory, pathogenic, or stress signals (Figure 1) [2,3]. The canonical NF-κB pathway is activated by several inflammatory cytokines, the most well-known being tumor necrosis factor alpha (TNFα). Stimulation of the TNF receptors, TLRs (Toll-like receptors), T-cell receptors, and interleukin receptors typically leads to the activation of this pathway and its downstream transcription factors, RelA/p65 and p50 [1]. The canonical NF-κB pathway is well characterized for having an inhibitor of kappa B kinase (IKK) complex consisting of IKKα, IKKβ, and IKKγ (also known as NEMO) [4]. IKKα and IKKβ are the catalytic units of the complex, and IKKγ is a regulatory subunit that is required for activation of IKKβ. After receptor activation, various kinases such as RIP1 (receptor interacting kinase 1), TAK1 (transforming growth factor beta-activated kinase 1), or TBK1 (TANK-binding kinase) phosphorylate and activate the IKK complex, preferentially on IKKβ [5]. Although constitutive proteasomal processing of p105 generates a subset pool of p50, p105 processing can also be induced through phosphorylation by IKKβ [6,7,8]. Activated IKKβ phosphorylates and induces the proteasomal degradation of IκBα (inhibitory kappa B alpha), which sequesters RelA-p50 complexes in the cytoplasm under basal, unstimulated conditions. Degradation of IκBα releases RelA-p50 complexes to translocate to the nucleus and regulate NF-κB-dependent gene transcription [9].

### 1.2. NIK and the Noncanonical NF-κB Pathway

**Figure 2:**
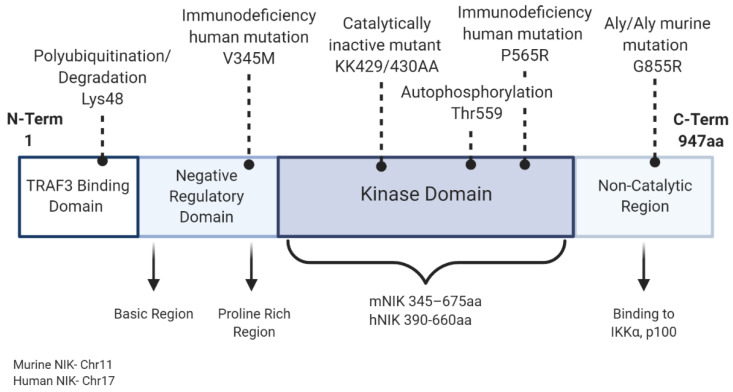
NF-κB-inducing kinase (NIK) structure and mutations. NIK is 947 amino acids long and consists of four main domains. The *Map3k14* gene is located on chromosome 11 in mice and 17 in humans. The first N-terminal domain mediates interaction between NIK and TRAF3 (TNF-receptor associated factor 3) to hold NIK inactive in the TRAF2/3/cIAPs (cellular inhibitor of apoptosis protein 1 and 2) complex. This first domain also contains a lysine (Lys48) whose ubiquitination mediates NIK degradation. The second domain consists of a negative regulatory domain (NRD) that regulates NIK’s C-term domain and its interaction with other proteins. The NRD consists of a basic leucine zipper and proline-rich repeat motifs. A novel mutation (V345M) found in an immunodeficient patient resides in this domain. The largest domain is the kinase domain, and its size varies from mouse to human orthologs. This domain contains sites for the well-characterized catalytically inactive mutant (KK429/430AA), autophosphorylation at Thr559, and another characterized point mutation found in immunodeficient patients (P565R). The final domain allows protein binding to IKKα and p100 and contains the point mutation (G855R) found to cause the immunodeficient phenotype in alymphoplasia mice.

**Figure 3:**
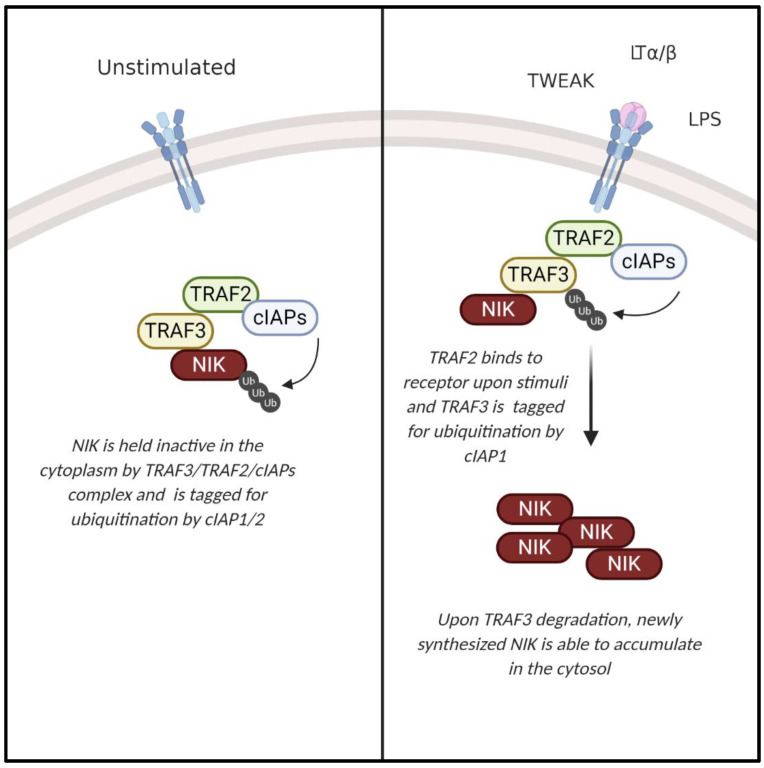
NF-κB-inducing kinase (NIK) activation. In an inactivated state, NIK is bound in a TRAF2/3/cIAPs (tumor necrosis factor receptor associated factor 2 and 3 and cellular inhibitor of apoptosis protein 1 and 2) complex where it is continuously tagged for ubiquitination. Once a receptor is bound and activated by extracellular stimuli, TRAF2 binds to the receptor and cIAP1 targets TRAF proteins for degradation. With TRAF3 degraded, newly synthesized NIK is able to accumulate in the cytoplasm.

The noncanonical NF-κB pathway ultimately leads to the activation of RelB-p52 dimers and is activated by a subset of tumor necrosis factor superfamily receptors (TNFSFRs), including TNFSFR12A (Fn14, Tweak receptor), lymphotoxin β receptor (LTβR), B-cell activating factor receptor (BAFF-R), receptor activator of NF-κB (RANK), CD40, and CD27 [10]. NF-κB-inducing kinase (NIK; gene name *Map3k14*) is a key upstream regulator of the noncanonical NF-κB signaling pathway [11,12]. Though initially identified by its ability to activate the canonical NF-κB pathway, loss of NIK did not impair TNF-induced activation of IKKβ/p65/p50, and NIK was subsequently shown to be essential for activation of noncanonical NF-κB signaling [13,14]. Regulation of NIK activity occurs primarily at the post-translational level. This serine/threonine kinase has four domains including a N-terminal region for TRAF3 binding, a negative regulatory domain (NRD), a core kinase domain, and a C-terminal domain responsible for binding with proteins such as IKKα and p100 (Figure 2) [15,16]. Under steady state conditions, NIK is bound to tumor necrosis factor (TNF) receptor-associated factor 2 and 3 (TRAF2/3) and cellular inhibitor of apoptosis protein 1 and 2 (cIAP1/2), resulting in its ubiquitination and continuous degradation. Stimulation with cytokines, such as CD40L, TWEAK (TNF-like weak inducer of apoptosis), LTα/β (lymphotoxin alpha/beta), or the endotoxin LPS (lipopolysaccharide), and specific binding to their membrane receptors sequesters TRAF2/3, which is then tagged for ubiquitination by cIAP1. Subsequent TRAF3 degradation allows accumulation of newly synthesized NIK within the cell (Figure 3) [1,2,3]. NIK stabilization and accumulation is critical for subsequent activation of the noncanonical NF-κB pathway [17]. Following receptor activation, NIK phosphorylates IKKα at Ser-176 and Ser-180 resulting in its activation and subsequent phosphorylation of p100 [18]. Phosphorylation of p100 induces binding of ubiquitin ligase, β-TrCP (beta transducin repeat containing proteins), and induces partial proteasomal processing to p52 [19,20]. Proteasomal processing of p100 removes the C-term ankyrin repeat domain, which functions similar to the inhibitory nature of mature IκB proteins, holding the noncanonical transcription factor RelB inactive in the cytoplasm. After processing of p100 into p52, p52-RelB translocates to the nucleus to regulate transcription [10] (Figure 1). Although IKKα is the key regulator of p100 phosphorylation and proteolytic processing, NIK functions to activate IKKα and also serves as an adaptor protein to recruit IKKα to p100. Ultimately, the NIK-IKKα-p100 complex is required for generation of mature, transcriptionally active p52 protein [20].

NIK activity is tightly regulated by several levels of negative regulatory feedback mechanisms. For example, activation of the noncanonical NF-κB pathway requires NIK and IKKα, but not NEMO. However, NEMO was found to suppress levels of NIK protein, suggesting a role for NEMO in limiting noncanonical NF-κB signaling [21]. TANK-binding kinase 1 (TBK1) directly interacts with NIK in a signal-dependent manner, inducing NIK phosphorylation on Ser-862, triggering degradation independently of TRAF3, and resulting in class switching to the immunoglobulin A (IgA) isotype [22,23]. Additionally, IKKα also functions to phosphorylate NIK at three residues in the C-terminus (Ser-809, Ser-812, and Ser-815), which are required for degradation of NIK downstream of BAFF-R and LTβR ligation [24]. Notably, IKKα-mediated negative regulation of NIK stability is independent of TRAF-cIAP complex, which limits basal levels of NIK in unstimulated cells. Similarly, the E3 ligase CRL4^DCAF2^ promoted the polyubiquitination and, consequently, the degradation of NIK in dendritic cells, independent of TRAF3 degradation [25].

## 2. NIK Regulation of Lymphoid Organogenesis, Immune Cell Development, and Hematopoiesis

NIK is best known for its regulation of the immune system development and function. It plays critical roles in both the development of lymphoid organs, as well as the function of different immune cells, including B-cells, T-cells, macrophages, dendritic cells, and hematopoietic stem cells (HSCs).

### 2.1. Lymphoid Organogenesis and B-Cell Development

The importance of NIK in lymphoid development was highlighted in the study of alymphoplasia (*Map3k14^aly/aly^*) mice, which lacked lymph nodes and Peyer’s patches [26]. This phenotype was linked to a spontaneous, autosomal recessive point mutation in the *Map3k14* gene that altered the C-terminal end of NIK, inhibiting its binding to proteins such as IKKα. In addition to lacking lymph nodes and Peyer’s patches, these mice displayed abnormal spleen and thymic structure with a reduction in regions of white blood cell housing specifically in the marginal zone of the spleen and medulla/cortex border of the thymus [14]. *Map3k14^aly/aly^* or complete NIK-knockout mice also exhibited stunted ability to mount an immune response with a reduction in B-cells and immunoglobulin development [13,14]. Maturation and viability of B-cells, particularly lymphocytes, are hindered, contributing to abnormal thymic and spleen structure. Many studies have shown that basal levels of immunoglobulins, specifically IgM and IgA, are found to be reduced in mice lacking NIK, and these mice are further unable to upregulate immunoglobulins in response to outside stressors [13,14]. Deletion of NIK in early B-cell development also produced a phenotype of inactive B-cells, particularly in peripheral tissues, further demonstrating the importance of NIK in all stages of B-cell development. The B-cell phenotype in NIK-knockout mice mimics that of BAFF (B-cell activating factor) or BAFF-R-knockout mice showing NIK is a critical downstream component of this signaling pathway to regulate B-cell development [27]. Moreover, consistent with murine models, dysregulation of immune function caused by mutations in NIK has also been identified in human patients with primary immunodeficiencies (see Section 3, below).

### 2.2. T-Cell Functions

In addition to regulating B-cells, loss of NIK also impacts T-cell maturation and activation. In T-cell-specific NIK-knockout mice, thymocytes underwent normal development, but NIK was observed to be critical for maintaining the homeostasis and function of peripheral T-cells [28]. Specifically, development of memory and regulatory T-cells were significantly reduced in the lymph nodes and spleen, reflected by an increase in naïve T-cells. NIK was shown to regulate the differentiation of T helper type Th1 and Th17 cells, as well as the recall responses of the effector T-cells to protein antigens in vivo [28]. Moreover, T-cell-specific NIK-knockout mice were resistant to the development of a T-cell-dependent autoimmune disease, experimental autoimmune encephalomyelitis [29]. T-cells lacking NIK exhibited reduced antigen responses, cytokine activation, and altered cytoskeletal dynamics compared to wild-type cells [29]. Indeed, reduced antiviral CD8^+^ T-cell immunity was observed in *Map3k14^aly/aly^* mice infected with lymphocytic choriomeningitis virus (LCMV) [30]. In another study, a T cell-intrinsic requirement for NIK was found in graft versus host disease (GVHD), whereby NIK-deficient T-cells transferred to major histocompatibility complex (MHC) class II mismatched mice failed to induce GVHD [31]. NIK has also been shown to have anti-inflammatory functions that are essential for Th2 cell function and is essential for protection from inflammatory disease arising from increased eosinophil accumulation in blood and tissues [32]. Interestingly, NIK-knockout mice spontaneously develop this hypereosinophilic-like disease independently of its phosphorylation and activation of IKKα [32]. Together these findings established NIK as an important cell-intrinsic mediator of T-cell functions in both immune and autoimmune responses.

### 2.3. Dendritic Cells

NIK was found to play a cell-intrinsic role in regulating intestinal homeostasis, promoting mucosal immunity against pathogens, as well as promoting chronic inflammation associated with inflammatory bowel disease (IBD) [33]. Although NIK was shown to be dispensable for dendritic cell development, a dendritic-cell-specific deletion of NIK altered the profile of commensal bacteria in the gut, specifically the segmented filamentous bacteria and *Enterococcus* spp. NIK promoted TLR-stimulated IL-23 production, which maintained an increased population of Th17 cells and innate lymphoid cells. These Th17 and innate lymphoid cells are major sources of IL-17, which, in turn, positively regulates IgA secretion and plays important roles in promoting the chronic inflammation associated with inflammatory bowel disease [33]. Interestingly, NIK signaling in dendritic cells was shown to be required for effector T-cell lineages [34], and impaired dendritic cell functions were observed in *Map3k14^aly/aly^* mice [35].

### 2.4. Macrophages

In addition to the aberrant development and paucity of B-cells, including marginal zone B-cells in *Map3k14^aly/aly^* mice, a lack of CD169^+^ macrophages was also observed, whereas other macrophage populations were not affected [30]. As macrophages are potent immunoregulatory cells of the innate immune system that fight pathogens, the loss of CD169^+^ macrophages was directly correlated with reduced immune activation in response to vesicular stomatitis virus (VSV) or lymphocytic choriomeningitis virus (LCMV) infection [30]. In another study, the sine oculis homeobox (SIX) homologue family transcription factors SIX1 and SIX2, which are developmentally silenced, were shown to function as negative regulators of noncanonical NF-κB signaling. Specifically, in differentiated macrophages, cytokine stimulation or pathogen infection resulted in reactivation of developmentally silenced SIX proteins by NIK-mediated suppression of the ubiquitin proteosome pathway. The net result of SIX1/2 reactivation was attenuation of inflammatory gene expression in response to persistent activation of the noncanonical NF-κB signaling cascade [36]. Together, these studies demonstrate that NIK plays important roles in regulating innate, as well as adaptive, immunity through macrophage functions in the pathogenesis of human disease.

### 2.5. Hematopoietic Stem Cells (HSCs)

Though normal under steady-state conditions, NIK-induced noncanonical NF-κB signaling has been shown to be essential for maintaining normal hematopoiesis, particularly in response to stressors, including excessive cytokines, chemotherapeutic agents, and hematopoietic transplantation [37]. The absence of NIK results in the dysregulation of hematopoietic stem/progenitor cell self-renewal and expansion capacity resulting from decreased proliferation and increased apoptosis. Therefore, disruption in normal inflammatory responses and the bone marrow microenvironment in NIK-deficient mice are major factors contributing to the development of bone marrow failure after stress [37].

## 3. NIK in Immune, Inflammatory, and Metabolic Diseases

Pathogen-sensing, immune system signaling, and metabolic signaling pathways are evolutionarily conserved and highly interconnected physiological processes. Indeed, as a central mediator of immune responses, aberrant NIK activation or expression has been linked to several immune and inflammatory, as well as metabolic diseases.

### 3.1. Immunodeficiency and Autoimmune Disorders

Defects in NIK and noncanonical NF-κB signaling are associated with severe immune deficiencies. Similar to *Map3k14^aly/aly^* mice, and other mouse models of NIK deficiency, mutations in NIK have been identified in patients with combined immunodeficiency [38,39]. Patients with homozygous recessive mutations in NIK (NIK^Pro565Arg^) displayed B-cell lymphopenia, decreased frequencies of class-switched memory B-cells, and impaired B-cell survival. Additionally, patients exhibited defects in both follicular helper and memory T-cells populations, as well as natural killer cells [38]. The NIK^Pro565Arg^ mutation resulted in impaired IKKα phosphorylation, and similarly, patients with a homozygous recessive NIK^Val345Met^ variant exhibited combined immunodeficiency, resulting from impaired NIK kinase activity [38,39].

### 3.2. Systemic Lupus Erythematosus

With regard to autoimmune disease, NIK was recently shown to be a potential therapeutic target for treatment of systemic lupus erythematosus (SLE) [40]. Inhibition of NIK using a NIK-specific small molecule inhibitor (NIK SMI1) increased survival in an experimental murine models of lupus by restricting overactive immune cells through a reduction in OX40, BAFF, and CD40 signaling [40].

### 3.3. Rheumatoid Arthritis

Rheumatoid arthritis (RA), another autoimmune disease characterized by chronic inflammation of the synovial joints, has also been linked to an increased NIK expression or activity. RA synovial fibroblasts secrete pro-inflammatory cytokines and growth factors, creating an abnormal microenvironment that supports activation of NIK and noncanonical NF-κB signaling that drive inflammation [41]. NIK was recently shown to promote inflammatory activation of human endothelial cells by RA synovial fluid and has previously been shown to promote inflammation-induced angiogenesis associated with RA endothelial cells [42]. In a GWAS study, NIK was found to be a genetic marker of RA susceptibility in different ethnic populations [43]. In another study of RA, significantly elevated NIK expression was observed in tertiary lymphoid organs, which are abnormal lymph node-like structures that form in peripheral tissues at sites of high chronic inflammation [44]. Similar to systemic lupus erythematosus, elevated BAFF signaling promotes the inflammatory RA microenvironment and antagonists have shown promising efficacy in the treatment of RA [41,45]. Therefore, as a downstream regulator of BAFF signaling, along with several other cytokine activated receptors, NIK is an apt target for RA therapeutics (see Table 1). Collectively, these data illustrate the nonredundant role for NIK in human immune responses, demonstrating that overactivation or loss-of-function mutations in NIK can cause multiple immune or inflammatory diseases.

### 3.4. Bone Disorders

Osteoporosis and generalized bone loss are characteristic features associated with both established and early rheumatoid arthritis [56]. RANK and RANK-ligand (RANKL) are key regulators of bone remodeling, and NIK has been shown to regulate osteoclast differentiation through RANK activation [57,58,59]. In one study, transgenic mice expressing constitutively active NIK showed a significant increase in osteoclast formation and increased bone erosion around the joints of the mice, suggesting that targeting NIK may also improve bone loss associated with RA [59]. Indeed, a small-molecule NIK inhibitor, Cpd33, was shown to inhibit osteoclastogenesis and bone resorption, preventing bone loss in ovariectomized mice [50].

### 3.5. Aging and Cardiovascular Disease

Aging, as well as associated increased inflammation and stress responses, is an important risk factor for several diseases. Recently, the FK506-binding protein 51 (FKBP51/FKBP5), a protein implicated in stress physiology, was found to significantly increase NIK-IKKα signaling to promote inflammation. Moreover, this correlation was strongly associated with increased risk of cardiovascular disease, specifically acute myocardial infarction, which is caused by chronic inflammation.

### 3.6. Metabolic Disorders

It has become increasingly evident that inflammation is a hallmark feature of metabolic disorders, including obesity and type 2 diabetes. Increased NIK expression has been linked to metabolic disorders associated with increased inflammation such as obesity, insulin resistance, and diabetes. Studies have shown that NIK is hyperactivated in livers of obese mice [60]. Overactivation of NIK in the liver of these obese mice led to glucose intolerance by inducing hyperglycemia through increased CREB-regulated glucagon activity [60]. Obesity-induced glucose intolerance by NIK was also observed in pancreatic β-cells leading to β-cell dysfunction and a decrease in insulin production [61]. Notably, tissue-specific deletion of NIK in the liver protected obese mice from liver inflammation and alcoholic steatosis. Consistent with that finding, pharmacological inhibition of NIK with B022 attenuated ethanol-induced hepatic steatosis and suppression of PPARα and fatty acid oxidation [46,62]. With regard to obesity, increased adiposity induces recruitment of pro-inflammatory M1 macrophages that are classically activated by LPS and INF-γ, and secrete inflammatory cytokines, most commonly TNFα [63]. Thus, stimulation of NF-κB is critical for macrophage activation and leads to subsequent elevation of NF-κB activation in surrounding cells, potentiating insulin resistance and type II diabetes [64]. Inhibition of NF-κB pathways, including NIK itself, is a promising therapeutic intervention to protect against diet-induced metabolic disorders [65].

## 4. Aberrant NIK Regulation in Cancer

Similar to other inflammatory diseases, cancer is marked by chronic inflammation and aberrant activation of immune signaling pathways. For example, cancerous cells recruit a variety of immune cells including T-cells and macrophages, resulting in elevated levels of proinflammatory cytokines that stimulate activation of NF-κB, promoting several hallmarks of cancer, including survival, proliferation, and metastasis [66]. Aberrant, constitutive activation of canonical NF-κB signaling is a feature of several cancers [67,68]. In these cases, although NF-κB typically functions as a tumor promoter, it can also function as a tumor suppressor [69]. In contrast to the canonical NF-κB pathway, which has been extensively studied in cancer, roles for NIK and the noncanonical NF-κB pathway in cancer are still emerging. Nevertheless, it is evident that similar to canonical NF-κB signaling, dysregulation of NIK and noncanonical NF-κB can lead to the formation and progression of several types of cancer, while in some cases, NIK suppresses tumorigenesis [70]. The initiation and progression of various cancers by NF-κB has been studied by dysregulation of individual subunits or of the pathways as a whole. Cancer models for NIK and the noncanonical NF-κB pathway have been analyzed at various stages from TRAF2/3 or cIAP degradation, to accumulation of NIK itself, to IKKα and its phosphorylation by NIK, or finally by increased expression or translocation of the downstream transcription factor RelB [71]. As with inflammatory and immune diseases, investigation of therapeutic effects of targeting NIK in cancer has been the focus of several studies (Table 1).

### 4.1. Cancer Stem Cells

Upregulation of NIK mRNA was shown to play an important role in maintaining the population of breast cancer stem cells (CSCs), which possess intrinsic properties of self-renewal and are important for tumor development, recurrence, and metastasis, particularly in the context of therapy resistance [72,73]. Knockdown of NIK reduced expression of breast CSC markers, reduced stem cell clonogenicity, and impaired tumorigenic potential in xenograft breast cancer mouse models [73]. Furthermore, a separate study demonstrated that specific deletion of RANK or IKKα in mouse mammary epithelial cells decreased tumorigenesis and invasive carcinoma [74]. In GBM initiating cells (GICs), it was found that NIK accumulation and noncanonical NF-κB activation was induced through epithelial V-like antigen (Eva1) degradation of TRAF2 and cIAP. This study further showed that the knockdown of RelB inhibited the self-renewal properties of human GICs and abolished tumorigenesis of mouse stem cells in vivo [75]. Increased expression of RelA, RelB, and IKKα was also observed in ovarian CSCs, and knockdown of IKKα decreased stem cell marker expression in those cells [76]. 

### 4.2. Leukemias and Lymphomas

As NIK has a crucial role in hematopoiesis, its dysregulation has been well studied in leukemias and lymphomas. Indeed, NIK inhibition has been shown to be a therapeutically efficacious treatment option for immune cell-derived cancers. For example, NIK and the noncanonical NF-κB pathway was shown to be stably activated in Hodgkin lymphoma (HL) cell lines and tumors, and treatment with a NIK small molecule inhibitor, 4H-isoquinoline-1,3-dione, significantly reduced HL cell viability [53]. In Mantel cell lymphoma (MCL), an aggressive B-cell malignancy that is refractory to treatment, NIK was identified as a new therapeutic target, particularly for lymphomas that are resistant to B-cell receptor pathway inhibitors [77]. On the other hand, studies in acute myeloid leukemia (AML) highlight the dual effects of regulating NIK stabilization as a therapeutic approach. AML cells, expressing a common gene fusion mutation in mixed-lineage leukemia 1 (MLL-ALL), exhibit constitutive activation of NIK and the noncanonical NF-κB pathway. Moreover, the stable knockdown of NIK increased the sensitivity of AML cells to chemotherapeutics and promoted apoptosis [78]. In contrast, a separate study demonstrated that activating NIK was beneficial in treating AML. Specifically, this group showed that the MLL-ALL fusion resulted in the stabilization of NIK and mice receiving hematopoietic stem cells transduced with MLL-ALL exhibited a significant delay in the initiation of AML due to increased NIK protein levels [79]. Notably, this myeloid-leukemia suppressive role of NIK differs from its tumor-promoting role in other lymphomas and functions to repress canonical NF-κB signaling, indicating that canonical and noncanonical NF-κB signaling may have opposing roles in AML.

Second mitochondria-derived activator of caspases (SMAC) mimetics target inhibitor of apoptosis proteins (IAPs) and are actively being studied for their chemotherapeutic potential through induction of apoptosis [80,81]. As cIAP1/2 are key ubiquitin ligases that bind NIK, use of SMAC mimetics increases NIK stabilization and activation [82]. As NIK stabilization can lead to canonical NF-κB activation and induction of apoptosis, SMAC mimetics have been used to treat several cancers including AML [83]. However, these effects are context dependent, and many cancer cells are resistant to SMAC-mediated apoptosis [84]. Similar to MML-ALL leukemias described above, in other cancers or cell types, NIK stabilization and activation by SMACs has been shown to protect cells from SMAC-induced death [85] (and see Section 4.5 below).

The use of SMAC mimetics is also being optimized in combinational therapies for treatment of tumors that take advantage of the immune checkpoint blockade. By expressing PD-L1, tumor cells are often able to evade cell death from surrounding T-cells. Upregulation of noncanonical NF-κB was seen in tumor dendritic cells, which increased IL-12 production, improving the synergistic relationship between dendritic cells and CD8^+^ T-cells. The priming of these T-cells upon noncanonical NF-κB activation and induction of IL-12 in neighboring dendritic cells improved T-cell sensitivity to aPD-1 treatment. This study further showed that the use of a cIAP antagonist on murine tumors increased invading dendritic cells and that chimera mice with NIK-deficient bone marrow had poor response to aPD-1 treatment [86]. Though this study highlighted the importance of the noncanonical NF-κB on the efficacy of tumor invading T-cells, further investigation is needed to understand the effects on the inactivation or overactivation of NIK on other tumor-invading immune cells such as macrophages. Furthermore, though separate studies identified NF-κB proteins, such as RelA, the downstream transcription factor of the canonical NF-κB pathway, as increasing cancer cell resistance to T-cell-induced death or increasing PD-L1 expression in ovarian cancer cells, little is known about the role of the noncanonical NF-κB pathway in regulating this immune checkpoint blockade in cancer cells [87,88].

### 4.3. Melanoma

Aberrant NIK activation has been observed in several solid cancer types, including melanoma, which is characterized by dysregulation of multiple signaling and tumor suppressor/oncogene pathways, such as *BRAF* [89]. For example, NIK expression and activity are increased by AKT and ERK signaling in melanoma cells compared with normal epidermal melanocytes, and knockdown of NIK reduced melanoma cell survival through reduction in noncanonical NF-κB pathway activation and expression of pro-survival genes [90,91]. Interestingly, mangiferin (1,3,6,7-tetrahydroxyxanthone-C2-β-D-glucoside), a natural bioactive agent with potent antioxidant, anti-inflammatory, and immunomodulatory effects, was recently identified as a NIK inhibitor [55]. Mangiferin and another NIK inhibitor, N-Acetyl-3-aminopyrazole, were shown to induce apoptosis and inhibit tumor growth and metastasis in a melanoma mouse model.

### 4.4. Breast Cancer

In addition to regulating breast cancer stem cells, elevated NIK expression was observed in breast carcinoma tissue vs. tumor-adjacent normal tissue surgically resected from human patients, and high NIK expression was associated with clinical stage and patient prognosis [92]. Recent gene co-expression network analysis identified NIK/NF-κB signaling pathway enrichment in chemotherapy-resistant breast carcinoma-associated fibroblasts [93]. However, another study found that expression of canonical and alternative NF-κB proteins are associated with improved relapse-free survival of breast cancer patients [94]. Taken together, these results highlight a critical need to understand how these pathways intersect in specific cellular and disease contexts.

### 4.5. Brain Cancer

Work from our group has elucidated tumor-promoting functions for NIK in glioma, including aggressive high-grade gliomas, such as glioblastoma, which are notoriously invasive and therapy resistant. We previously demonstrated that NIK promotes glioma invasion through TWEAK and noncanonical NF-κB activation, which induced expression of MMP9 (matrix metalloprotease 9). NIK also induced matrix metalloproteinase 14 (MT1-MMP) upregulation in invadopodia and promoted glioma cell invasion in a manner that was dependent on noncanonical NF-κB, but independent of canonical NF-κB signaling [95]. Additionally, overexpression of NIK significantly induced tumor cell invasion and increased tumor size [96]. Subsequently, we showed that NIK can regulate cancer cell invasion through regulation of mitochondrial dynamics and mitochondrial trafficking at the leading edge of migrating glioma cancer cells [97]. With regard to therapeutics, although the SMAC mimetic and IAP agonist BV6 has demonstrated potential for inducing cell death in a large number of cancers, it was shown to induce accumulation of NIK, resulting in increased cell elongation, migration, and invasion in primary glioblastoma cells [85]. Together with our findings that TWEAK treatment induced NIK expression and promoted NIK-dependent glioblastoma cell invasion, these results suggest that therapy-induced inhibition of NIK should be considered as a potential determinant of treatment efficacy in the context of glioblastoma.

### 4.6. Other Solid Tumors

NIK overexpression has been observed in human pancreatic cancer samples, where TRAF2 degradation increased NIK stabilization [98]. Furthermore, NIK overactivation was observed to have a role in cell survival and proliferation in pancreatic cancer cell lines, implicating NIK as therapeutic target for growth inhibition [99]. In a human study of patients biopsied with gastric cancer, NIK was not only highly expressed in tumor tissue but it was found to be significantly associated with tumor differentiation and had a higher positive rate in later stages of the cancer [100].

## 5. NF-κB-Independent Roles for NIK in Disease

**Figure 4:**
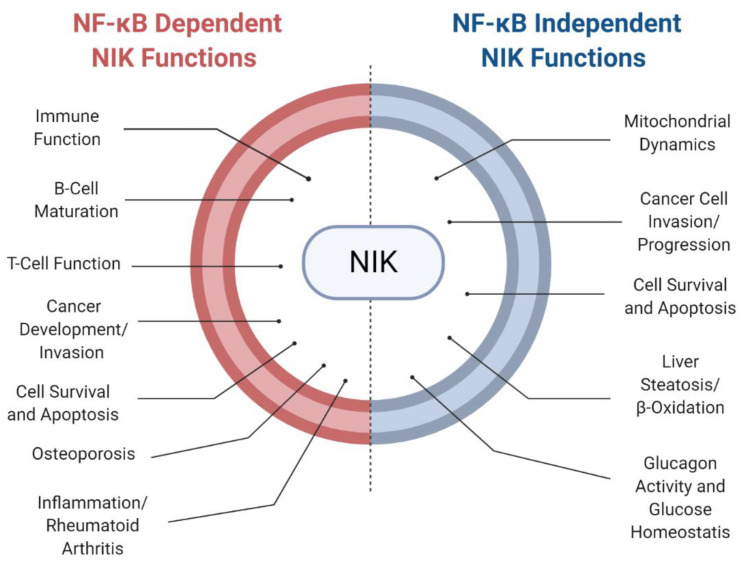
NIK mediation of health and disease. NIK’s role in NF-κB-dependent and -independent regulation of cellular processes and disease.

Finally, several studies highlighted here have observed NF-κB-independent NIK functions in regulating cellular processes and disease (Figure 4). As previously discussed, NIK induction of liver steatosis was observed through regulation of PPARα phosphorylation and recruitment of ERK1/2 and MEK1/2. NF-κB-independent roles for NIK were observed in the regulation of beta oxidation and inflammation in the liver [46]. NIK was also observed to induce apoptosis independent of the noncanonical NF-κB pathway through phosphorylation of RIP1 [101]. Additionally, NIK independently regulates glucose homeostasis by direct phosphorylation of CREB, increasing its stabilization to regulate glucagon activity [60]. In cancer models, increased NIK activity has been linked to cell survival and tumorigenesis through association with β-catenin and upregulation of pro-survival genes [91]. Furthermore, NIK can regulate mitochondrial function through dynamin-related protein 1 (DRP1) activity, in a manner that is independent of downstream IKK/NF-κB signaling, resulting in amplification of tumorigenic effects and deregulated cancer metabolism [97]. These studies present a strong rationale for specific therapeutic targeting of NIK in various inflammatory diseases and cancer.

## 6. Conclusions

While much remains to be learned about the full spectrum of NIK’s functions in health and disease, particularly with regard to NF-κB-independent signaling, it is evident that NIK and noncanonical NF-κB signaling are important for many diverse functions of immune cells, inflammatory disease pathogenesis, and malignancies. Although the dual roles that NIK plays in promoting or attenuating cell survival depending on cell context must be an important consideration in therapeutic approaches targeting NIK, small-molecule pharmacological inhibitors of NIK have tremendous potential to be potent stand-alone or combinatorial therapeutics in a wider range of diseases than previously appreciated.

## Figures and Tables

**Table 1:** Nuclear factor kappa-light-chain-enhancer of activated B cells-inducing kinase (NIK) inhibitors. Brief list of small molecule and natural NIK inhibitors and correlating disease treatment.

Disease	NIK Inhibitor	Therapeutic Effect
**Inflammatory/immune diseases:** Liver inflammation/steatosis Rheumatoid arthritis Osteoporosis Lupus	**B022**	Inhibitor of NIK and subsequent liver inflammation and steatosis under alcoholic liver model [46]. Reduction in liver inflammation and damage due to toxin treatment of carbon tetrachloride [47].
	**XT2**	NIK inhibitor that suppresses hepatocyte inflammation generated by toxin-induced liver injury by carbon tetrachloride, but to a lesser extent than B022 [48].
	**NIK SMI1** **(small molecule inhibitor 1)**	Favorable to inhibition of BAFF-induced B-cell survival [49]. Inhibits NIK in immune cells with improved survival rate in murine model of lupus [40].
	**Cpd33** **(NIK Specific Inhibitor Compound 33)**	Specifically inhibits noncanonical NF-κB pathway. Studied with in vitro RANKL activation and inhibition of downstream transcription factor NFATc1. Treatment with Cpd33 inhibits osteoclastogenesis in vitro and in murine OVX model. Cpd33 treatment also inhibited bone absorption ability in mature osteoclasts and overall prevented bone loss in murine model [50].
**Cancer:** Leukemias Lymphomas Pancreatic cancer Breast cancer Melanoma	**N-(3-(6-benzamido-3a,7a-dihydrobenzo[d]oxazol-2-yl)-phenyl)benzamide**	NIK inhibitor with general IC_50_ of 48.9 μM and inhibition rate of about 56%. This is inhibitor was less efficient than B022 that had an IC_50_ of 9.9 nM. In SW1990 (pancreatic cancer cells), inhibitor had IC_50_ of 20.1 μM [51].
	**4H-isoquinoline-1,3-dione**	Inhibits NIK activity by insertion to ATP-binding site. Has an IC_50_ of 51 μM and analogs are inhibitors for CDK4 and IGF-1R [52]. Treatment of Hodgkin lymphoma through inhibition of NIK/RelB [53].
	**N-Acetyl-3-aminopyrazoles**	Selective inhibitor of NIK with IC_50_ of about 8.4 μM. Over 80% NF-κB inhibition in multiple myeloma cells at 25 μM, but low inhibition in MDA-MB-231 and SKBr3 cells even at 100 μM [54].
	**Mangiferin**	A natural inhibitor of NF-κB kinases including NIK in a dose-dependent manner. Treatment with 100–200 mg/kg of mangiferin significantly inhibited melanoma tumor growth and metastasis [55].

## Abbreviations

ALL	acute lymphoblastic leukemia
AML	acute myeloid leukemia
Aly	alymphoplasia
BAFF/BAFFR	B cell activating factor/B cell activating factor receptor
β TrCP	beta transducin repeats containing proteins
CDK4	cyclin-dependent kinase 4
cIAP1/2	cellular inhibitor of apoptosis protein 1 and 2
CML	chronic myeloid leukemia
Cpd33	compound 33
CREB	cAMP response element binding
CSC	cancer stem cells
DRP1	dynamine-related protein 1
ERK	extracellular signal regulated kinase
Eva1	epithelial V-like antigen 1
FKBP5	FK506-binding protein 51 (FKBP51)
GBM	glioblastoma multiforme
GIC	GBM initiating cells
GVHD	graft versus host disease
GWAS	genome-wide association study
HL	Hodgkin lymphoma
hNIK	human NIK
IC_50_	inhibitory concentration (half of maximal)
IGF-1R	insulin like growth factor 1 receptor
IBD	inflammatory bowel disease
IκBα	inhibitory kappa B alpha
IKKα	inhibitory kappa B kinase alpha (aka CHUK)
IKKβ	inhibitory kappa B kinase beta
IKKγ	inhibitory kappa B kinase gamma (aka NEMO)
IL	interleukin
INF-γ	interferon gamma
LCMV	lymphocytic choriomeningitis virus
LPS	lipopolysaccharide
LTα/β	lymphotoxin alpha/beta
MAP3K14	mitogen activated protein kinase, kinase, kinase 14 (aka NIK)
MCL	Mantle cell lymphoma
MHC	major histocompatibility complex
MLL1	mixed-lineage leukemia 1
MMP9	matrix metalloproteinase 9
mNIK	mouse NIK
MT1-MMP	membrane-type matrix metalloproteinase
NFATc1	nuclear factor of activated T-cells 1
NF-κB	nuclear factor kappa-light-chain-enhancer of activated B cells
NIK	NF-κB-inducing kinase
NRD	negative regulatory domain
OVX	ovariectomy
PD1	programmed cell death protein 1
PPARα	peroxisome proliferator-activated receptor alpha
RA	rheumatoid arthritis
RANK	receptor activator of NF-κB
RANKL	receptor activator of NF-κB ligand
RIP1	receptor interacting kinase 1
SIX	sine oculis homeobox homologue family transcription factors
SMAC	second mitochondria-derived activator of caspases
SMI1	small molecule Inhibitor 1
SPP	several species
TAK1	transforming growth factor beta-activated kinase 1
TBK1	TANK-binding kinase 1
TLR	Toll-like receptor
TNF	tumor necrosis factor
TNFSFR	tumor necrosis factor superfamily receptor
TRAF2/3	TNF-receptor associated factor 2 and 3
TWEAK	tumor necrosis factor-like weak inducer of apoptosis
VSV	vesicular stomatitis virus
